# Autoimmune Diseases Affecting Hemostasis: A Narrative Review

**DOI:** 10.3390/ijms232314715

**Published:** 2022-11-25

**Authors:** Emmanuel J. Favaloro, Leonardo Pasalic, Giuseppe Lippi

**Affiliations:** 1 Haematology, Institute of Clinical Pathology and Medical Research (ICPMR), Sydney Centres for Thrombosis and Haemostasis, NSW Health Pathology, Westmead Hospital, Westmead, Sydney, NSW 2145, Australia; 2 School of Dentistry and Medical Sciences, Faculty of Science and Health, Charles Sturt University, Wagga Wagga, NSW 2678, Australia; 3 School of Medical Sciences, Faculty of Medicine and Health, University of Sydney, Westmead Hospital, Westmead, Sydney, NSW 2145, Australia; 4 Westmead Clinical School, University of Sydney, Westmead, Sydney, NSW 2006, Australia; 5 Section of Clinical Biochemistry, University of Verona, 37129 Verona, Italy

**Keywords:** autoimmune disease, hemostasis, thrombosis, bleeding, COVID-19, acquired hemophilia, antiphospholipid (antibody) syndrome (APS), lupus anticoagulant, heparin induced thrombotic thrombocytopenia, vaccine induced (immune) thrombotic thrombocytopenia, immune thrombocytopenia, immune thrombotic thrombocytopenia

## Abstract

Hemostasis reflects a homeostatic mechanism that aims to balance out pro-coagulant and anti-coagulant forces to maintain blood flow within the circulation. Simplistically, a relative excess of procoagulant forces can lead to thrombosis, and a relative excess of anticoagulant forces can lead to bleeding. There are a wide variety of congenital disorders associated with bleeding or thrombosis. In addition, there exist a vast array of autoimmune diseases that can also lead to either bleeding or thrombosis. For example, autoantibodies generated against clotting factors can lead to bleeding, of which acquired hemophilia A is the most common. As another example, autoimmune-mediated antibodies against phospholipids can generate a prothrombotic milieu in a condition known as antiphospholipid (antibody) syndrome (APS). Moreover, there exist various autoimmunity promoting environments that can lead to a variety of antibodies that affect hemostasis. Coronavirus disease 2019 (COVID-19) represents perhaps the contemporary example of such a state, with potential development of a kaleidoscope of such antibodies that primarily drive thrombosis, but may also lead to bleeding on rarer occasions. We provide here a narrative review to discuss the interaction between various autoimmune diseases and hemostasis.

## 1. Introduction

**Figure 1:**
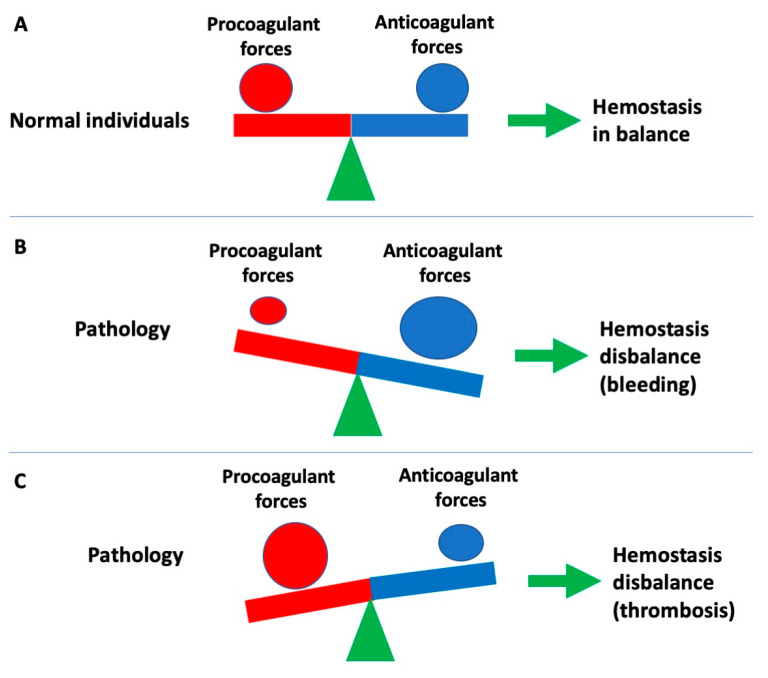
Hemostasis can be considered as reflecting a balance of procoagulant and anticoagulant forces. (**A**) In normal individuals, these forces are in balance, and this acts to prevent overt bleeding or thrombosis. (**B**) In some pathological states, a relative reduction in procoagulant forces or an excess of anticoagulant forces, can lead to a hemostasis disbalance and bleeding. (**C**) In some pathological states, an excess of procoagulants forces, or a relative reduction in anticoagulant forces, can lead to a hemostasis disbalance and thrombosis.

Hemostasis reflects a homeostatic physiological mechanism that aims to balance out procoagulant and anticoagulant forces to maintain blood flow within the circulation (Figure 1). When there is a relative excess of procoagulant forces, then thrombosis can ensue; alternatively, a relative excess of anticoagulant forces (or a relative reduction in procoagulant forces) can lead to pathological bleeding (Figure 1). Moreover, there are both natural procoagulants and anticoagulants that interplay to maintain normal physiological hemostasis (Table 1). There are a wide variety of congenital disorders associated with bleeding or thrombosis, including hemophilia, and von Willebrand disease (VWD), as well as thrombophilic conditions caused by deficiency of natural anticoagulants or presence of clotting factor (F) variants (FV Leiden, Prothrombin G20210A) associated with thrombosis. In addition, there exist a wide variety of autoimmune diseases that can also lead to hemostasis dysfunction, and thus either to a bleeding or thrombosis phenotype; these may sometimes also mimic congenital disorders of hemostasis (Table 2). For example, auto-immune mediated antibodies generated against clotting factors can lead to bleeding, of which acquired hemophilia A (antibodies against FVIII) is the most common [1,2,3,4,5,6]. Acquired von Willebrand syndrome (AVWS) represents an additional example of an acquired bleeding disorder [7,8,9,10,11], sometimes associated with antibodies against von Willebrand factor (VWF), an adhesive plasma protein that otherwise facilitates attachment and immobilization of blood platelets to the sites of vascular injury, thereby promoting platelet plug formation. Alternatively, auto-immune mediated generation of antibodies against various hemostasis components can promote a prothrombotic milieu. For example, antibodies generated against phospholipids can lead to a condition known as antiphospholipid (antibody) syndrome (APS) [12,13,14,15]. Additional prothrombotic conditions can arise through generation of antibodies against platelet factor 4 (PF4), including heparin-induced thrombotic thrombocytopenia (HITT) and vaccine-induced (immune) thrombotic thrombocytopenia (VITT) [16,17,18,19,20]. In this narrative review, we discuss the interaction between various autoimmune diseases and hemostasis, that can lead to acquired disorders of hemostasis, and either bleeding or thrombosis.

## 2. A Brief Overview of Hemostasis

**Figure 2:**
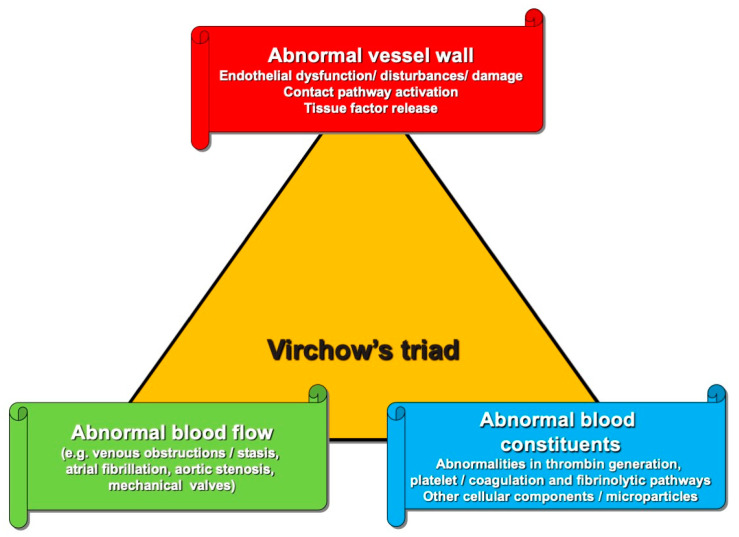
Virchow’s triad. Thrombosis can occur due to the disruption of any of the three components listed.

As noted, physiological hemostasis acts to maintain blood flow in the circulation, and thereby prevent bleeding or thrombosis. Hemostasis normally reflects a balance of procoagulant and anticoagulant forces (Figure 1), reflecting an equilibrium of procoagulant blood components with anticoagulant blood components (Table 1). Another popular way to look at perturbed hemostasis leading to a procoagulant state and possible thrombosis is through the Virchow’s Triad (Figure 2), whereby intravascular vessel wall damage, stasis of blood flow, and/or the presence of hypercoagulability reflect key ingredients. Hemostasis actually reflects the interaction of multiple processes, which can conventionally and conveniently be separated into ‘primary hemostasis’, ‘secondary hemostasis’ and ‘fibrinolysis’ [21,22,23]. However, this separation, whilst convenient for the laboratory aided investigation of hemostasis dysfunction, does not truly represent the in vivo hemostatic system, in which all these processes interact at multiple points and merge at several levels. Nevertheless, investigation of hemostasis dysfunction may require evaluation of level and activity of a variety of hemostasis components, including those listed in Table 1. Hemostasis dysfunction leading to bleeding or thrombosis can occur at any point in these hemostasis pathways, and due to the dysfunction of any of the contributing components. However, some components seem to be more important than others, in so far as their dysfunction more commonly leads to breakdowns in hemostasis, and thus bleeding or thrombosis.

**Figure 3:**
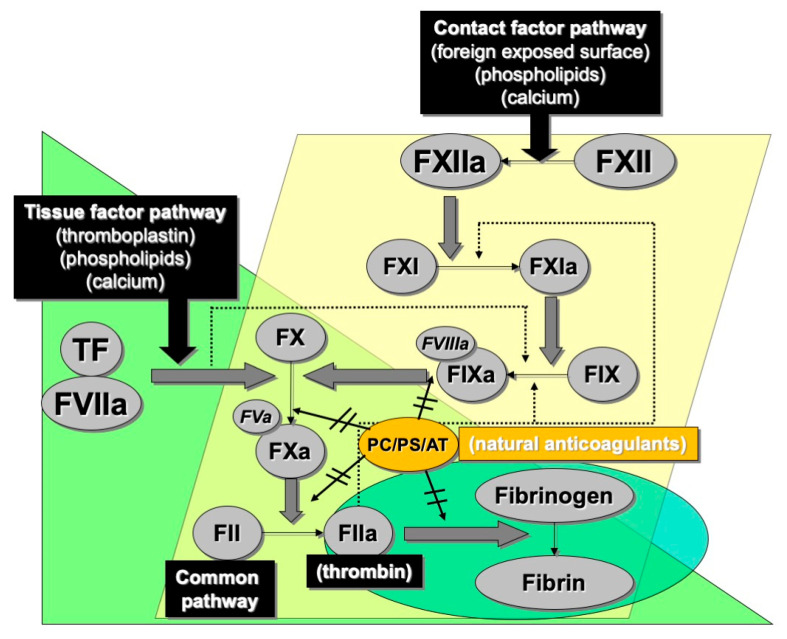
The historical cascade model of hemostasis reflects a series of protease-led activation of subsequent factors in the cascade, and closely reflects the routine laboratory tests we use to broadly assess coagulation. The ‘contact factor pathway’ (also historically known as the intrinsic pathway of coagulation) essentially matches the activated partial thromboplastin time (APTT) test, the ‘tissue factor pathway’ (also historically known as the extrinsic pathway of coagulation) essentially matches the prothrombin time (PT) test. Both converge into a so-called common pathway, which ultimately leads to generation of thrombin (activated factor II), and conversion of the most abundant coagulation plasma protein (soluble) fibrinogen to (insoluble) fibrin. A series of natural anticoagulants (protein C [PC], protein S [PS] and antithrombin [AT] help to control the coagulation process.

**Figure 4:**
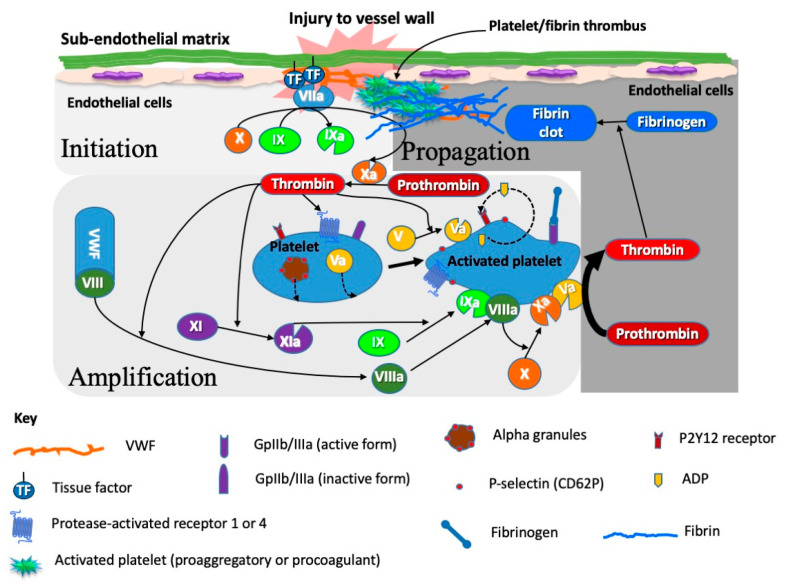
The cell based or platelet-driven model of hemostasis reflects the incorporation of platelets and other components of primary hemostasis (e.g., plasma and platelet derived VWF) and the secondary elements of hemostasis (i.e., clotting factors) to provide a composite hemostasis process. Platelets provide both the building blocks to the formation of the platelet plug (or thrombus) as well as the assembly point for secondary hemostasis pathways. Platelets contain various granules which release their cargo upon platelet activation; this cargo comprises procoagulant material such as factor V, fibrinogen, and VWF.

In brief, primary hemostasis is reflected by the activity of platelets and various hemostasis proteins that bind to platelets and help to immobilize these blood elements to sites of tissue damage, thereby causing platelet activation, and local platelet-mediated release of procoagulant proteins that amplify their attachment and stimulate the eventual formation of the platelet ‘plug’, which at the end acts to seal the damage and prevent excessive blood loss. Secondary hemostasis comprises a variety of clotting proteins, classically depicted by the coagulation cascade (Figure 3), although this ‘cascade’ better reflects the in vitro tests that we use to interrogate disturbances of secondary hemostasis, rather than what actually occurs in vivo, which is instead better reflected by the cell-based model of hemostasis (Figure 4). The primary and secondary hemostasis pathways are intertwined. For example, platelet activation in the primary hemostasis pathway leads to release of secondary hemostasis proteins, including FV, fibrinogen and VWF, and also provides a surface upon which secondary hemostasis proteins can assemble and potentiate clot formation. Additionally, platelet activation permits binding of some of those proteins, especially VWF and fibrinogen, to help form the stable platelet plug. The VWF and fibrinogen attach to platelets and to damaged subendothelium, to provide a scaffold for assembly of the platelet plug, and VWF is also able to release its own cargo of FVIII locally, to further promote secondary hemostasis. The third component, fibrinolysis, acts to break down the platelet plug, and prevent excessive thrombus formation, and eventually permit wound repair.

## 3. Autoimmune Disorders Leading to Bleeding

Autoimmune disorders that lead to bleeding essentially reflect generation of autoantibodies directed against procoagulant components of hemostasis. This is different to the typical development of alloantibodies in patients deficient in certain coagulation factors who receive replacement therapy. Alloantibodies will occur in a large proportion of hemophilia A (FVIII deficiency) and B (FIX deficiency) at some point in their treatment lives [24,25,26,27], since the coagulation proteins they are unable to produce naturally may be seen as ‘foreign’ by the immune system when these are provided as replacement products. Albeit less often, autoantibodies can also occur spontaneously in patients who do not have natural deficiencies, but who instead acquire a deficiency due to some disease process or even in circumstances of dysregulation of the immune system. In these settings, the development of autoantibodies against naturally occurring proteins is triggered by certain event (usually encompassing a strong stimulation of the immune system by infections, inflammation, pathologies like cancer or certain therapies) at a certain point of the patient’s life.

### 3.1. Acquired Hemophilia A

Acquired hemophilia A is the most common acquired bleeding disorder that develops from such antibodies, encompassing the generation of anti-FVIII antibodies [1,2,3,4,5,6] (Table 2). The majority of these antibodies inhibit the function of the clotting factor (hence the term ‘inhibitors’ is often used), meaning that they interfere with normal FVIII activity (e.g., binding to VWF or phospholipid surfaces). This leads to a state in which FVIII is unable to function properly, thereby blocking the hemostasis pathways that depend on FVIII (see Figure 3 and Figure 4). The hemostasis laboratory evaluates for the presence of these functionally inhibiting antibodies using the Bethesda assay, which can quantify the level of inhibition, and also predicts the amount of replacement FVIII required to overcome the antibody present [1,28]. Patients with low levels of FVIII inhibitors (generally ≤5 Bethesda Units [BU]/mL) can usually be successfully treated with FVIII replacement therapy. Those with higher levels of FVIII inhibitors (generally >5 BU/mL) may require FVIII bypassing agents, as well as immunosuppression therapy (rituximab, corticosteroids alone or in combination with cyclophosphamide) to eliminate the autoantibodies. Some antibodies against FVIII are not functionally inhibiting and instead act to clear FVIII from the circulation. There are several autoimmune conditions associated with generation of anti-FVIII autoantibodies, including systemic lupus erythematosus (SLE), rheumatoid arthritis, multiple sclerosis, Sjogren syndrome, and temporal arteritis. Other conditions that may lead to development of these inhibitors include inflammatory bowel disease; infections; diabetes; hepatitis; respiratory or dermatological diseases; blood (hematological) malignancies or certain solid cancers. An association with use of certain drugs, such as penicillin or interferon, and with pregnancy has also been reported, mainly in the post-partum period. Affected individuals develop complications associated with abnormal, uncontrolled bleeding into the muscles, skin and soft tissue that can occur spontaneously, during surgery or following trauma. Specific symptoms can include nosebleeds (epistaxis), bruising throughout the body, solid swellings of congealed blood (hematomas), blood in the urine (hematuria) and gastrointestinal or urogenital bleeding. Intracranial hemorrhage is rare, but can be fatal. The estimated prevalence of acquired hemophilia A is around 1 in 1,000,000 individuals/year.

A specific mention deserves attention, being recently identified acquired hemophilia A following coronavirus disease 2019 (COVID-19) vaccination. Although a causality link between vaccine and acquired hemophilia A has not been definitely ascertained, several of such cases have already been reported [29,30,31,32,33]. A possible explanation is that vaccines may foster or boost immune reactions, followed by insurgence of new autoimmune events or exacerbation of pre-existing but latent immune dysregulations, which can ultimately drive the clonal selection of B cells producing specific autoantibodies, including those against FVIII.

### 3.2. Other Acquired Bleeding Disorders Involving Clotting Factors

Although acquired hemophilia A represents the most common acquired factor defect, antibodies can also arise to all other clotting factors [34], of which FV is perhaps best known [35,36,37,38]. In the case of FV, the generation of most historical causes of acquired FV deficiency were due to the use of bovine thrombin and fibrinogen in some surgeries to help seal wounds (called ‘fibrin glue’). Unfortunately, the bovine thrombin used was often ‘contaminated’ with trace amounts of bovine FV, leading to generation of anti-bovine FV antibodies that cross reacted with human FV [38]. Up to 200 patients with autoimmune FV deficiency have been identified in Japan, where a national-wide Collaborative Research Group operates [35]. These authors identified a relatively mild type of bleeding diathesis, associated with lower mortality rate than that previously reported in the literature for both autoimmune FV deficiency and other autoimmune clotting factor inhibitors. The Japanese cohort showed variable FV inhibitor titres and both neutralizing and non-neutralizing autoantibodies. Although spontaneous resolution occurs in some patients, timely initiation of hemostatic and immunosuppressive therapies helped arrest the bleeding and eliminate anti-FV antibodies, resulting in a high cumulative recovery rate. The authors advised the importance of immunological anti-FV antibody detection to avoid missing cases presenting with non-neutralizing anti-FV autoantibodies. Another study from the USA identified 8 cases of autoimmune FV inhibitors in a single institution (the Mayo Clinic) over a period of 18 years [36]. Overall, 2/8 (25%) patients experienced no bleeding, and there was no correlation between inhibitor titres and/or FV activity levels and clinical bleeding. Hemostatic management included fresh frozen plasma, platelet transfusion, activated prothrombin complex concentrates, and recombinant activated FVII (rFVIIa). Only 2 patients received immunomodulatory treatment. Their clinical cohort confirmed the variable clinical phenotype for individuals with autoimmune FV deficiency and supports the notion that management should be guided by clinical symptoms and not FV activity or inhibitor titre.

Acquired deficiencies or defects of other clotting factors have also been described, but most acquired defects do not have an autoimmune basis [34,39,40,41,42,43]. For example, FX deficiency can occur due to adsorption of FX to amyloid fibrils in patients with amyloidosis. Additionally, vitamin K deficiency will lead to a relative deficiency of the clotting factors that depend on vitamin K (i.e., factors II, VII, IX and X) for full functionality [39]. Liver disease is another cause of multiple factor deficiency, since most clotting factors are produced in the liver [40]. Nevertheless, auto-immune related factor deficiencies have been reported for essentially all the other clotting factors [34]. In addition, acquired autoimmune FXIII deficiency has also been identified [44,45,46].

### 3.3. Acquired Von Willebrand Syndrome (AVWS)

Another somewhat common acquired bleeding disorder arises due to defects in, or reduction of, the adhesive plasma protein VWF, and this is conventionally termed AVWS [7,8,9,10,11]. In contrast to acquired hemophilia A, which can usually be shown to have a functionally inhibiting autoantibody basis, most forms of AVWS are not autoimmune related, and even if an autoantibody is identified, it is usually not functionally inhibiting. The most commonly identified forms of AVWS nowadays are represented by structural cardiac defects (e.g., aortic valvular stenosis) or due to presence of ‘foreign surfaces’ (e.g., left ventricular assist devices) [47,48,49,50,51]. These defects/surfaces lead to AVWS by causing absorption or loss of high molecular weight (HMW) VWF, which is otherwise the most functionally active form of VWF, and which can facilitate more platelet adhesion to damaged tissue during the hemostasis process. Additional common mechanisms of AVWS include absorption of VWF on malignant cells, reduced production (e.g., as in hypothyroidism), and increased blood platelet count such as in essential thrombocythemia. Nevertheless, a proportion of AVWS cases do have an autoimmune basis (e.g., arise in autoimmune disorders such as SLE, scleroderma, and APS), or else are associated with production of autoantibodies against VWF [52,53].

However, unlike acquired hemophilia A, where a functionally inhibiting autoantibody is likely to be present, most causes of immune AVWS lead to clearance of VWF rather than inhibiting its function. Thus, most forms of AVWS do not show the presence of functionally inhibiting antibodies, and Bethesda like assays tend to be negative. Although VWF-clearing antibodies may be present in AVWS, these are difficult to prove. Laboratories can attempt to assess the presence of antibodies against VWF using ELISA (enzyme linked immunosorbent assays), but the high risk of false positive test results using these assays, and their technical complexity, prevents their widespread use. Instead, antibody-mediated clearance of VWF can be inferred following a pharmacodynamic response to an infusion of VWF, with rapid clearance evident, and in the absence of alternate reasons for rapid clearance. As indicated, rapid clearance of VWF can alternatively arise in the presence of some malignancies, where VWF is absorbed onto cancer cells, as well as some congenital disorders (e.g., VWD of type 1 ‘clearance’). The bleeding phenotype in AVWS is similar to that of congenital VWD, being mostly mucocutaneous in nature. Several hundred cases of AVWS have been reported in the medical literature to date, but numbers may be higher because of under-reporting, or misreporting as congenital VWD. The number of autoimmune AVWS cases is not known, although a recent assessment indicated these can be identified, providing clinicians and laboratories perform the appropriate investigations [53]. Treatment of AVWS depends on the underlying cause of the condition, but if an immune basis is identified, treatment could be supplemented with immunosuppression therapy.

### 3.4. Acquired Autoimmune Thrombocytopenia (ITP)

Another autoimmune condition that causes bleeding is acquired immune thrombocytopenia (ITP) [54,55]. ITP affects approximately 1 in 20,000 people. Patients typically present with clinically benign mucocutaneous bleeding, but morbid internal bleeding can also occur. Diagnosis remains primarily clinical in nature, possibly only after ruling out other causes of thrombocytopenia through clinical history and laboratory testing. Many adult patients do not require treatment. For those requiring intervention, initial treatment of adult ITP is with corticosteroids, intravenous immunoglobulin, or intravenous anti-RhD immune globulin [54,55]. Further extensive discussion of ITP is beyond the scope of the current review, and would warrant a separate review. Readers are referred to international guidelines on this topic [56], which detail the investigation and management of primary immune thrombocytopenia.

## 4. Autoimmune Disorders Leading to Thrombosis

### 4.1. Antiphospholipid Antibody Syndrome (APS)

As already briefly mentioned, antibodies can develop against various phospholipids and give rise to APS, an auto-immune condition associated both with thrombosis and pregnancy/fetal morbidity/mortality [12,13,14,15,57]. These autoantibodies can be detected in the laboratory using either ‘solid phase’ assays (by ELISA or chemiluminescence), as well as liquid phase (or clot-based) assays for the so-called lupus anticoagulant (LA) [58,59,60]. The ‘solid phase’ antiphospholipid antibody (aPL) assays include ‘anti-cardiolipin’ (aCL), ‘anti-beta-2-glycoprotein I’ (aβ2GPI), ‘anti-prothrombin’ (aPT), ‘anti-phosphatidylserine’ (aPS), or complexes (such as ‘anti-phosphatidylserine/prothrombin’ [aPS/PT] complex). Within the diagnosis of APS, the titre (or strength) of the antibodies detected, as well as the number of different activities (e.g., double positive or triple positive) align to the thrombotic risk profile. The clot-based assay approach for LA requires the performance of various clotting assays based on different principles and using different levels (low and high) of phospholipids to increase or reduce sensitivity to LA [58,60]. Unfortunately, although LA tends to be more highly associated with adverse events in APS than solid phase assays, LA tests are also very sensitive to many clinical anticoagulants, which may be given to patients to treat or prevent thrombosis, but when present can lead to false positive LA findings [61]. To further complicate the clinical diagnosis and management of APS, some patients have clearing antibodies against prothrombin, which affects the baseline prothrombin time (PT) and international normalised ratio (INR), with affected patients having a possible risk of bleeding; this condition is termed “Lupus anticoagulant-hypoprothrombinemia syndrome” by some workers in the field [62].

### 4.2. Heparin Induced Thrombotic Thrombocytopenia (HITT), and HITT-like Syndromes (Including Vaccine Associated (Immune) Thrombotic Thrombocytopenia (VITT))

Another well-known autoimmune condition associated with thrombosis is the so-called heparin induced thrombotic thrombocytopenia (HITT; sometimes abbreviated HIT) [16,17,63,64]. This arises in a small proportion (~1%) of patients who are treated with heparin anticoagulant. In susceptible patients, heparin forms a complex with PF4, a platelet release product, and antibodies form against the complex. These antibody/PF4/heparin complexes bind to platelets via the Fc receptor, causing platelet activation, release of platelet contents, formation of platelet microparticles, and thus further fuelling thrombus formation in both the venous and the arterial circulation. It is a conundrum that provision of heparin, aiming to prevent thrombosis, in some patients instead leads to thrombosis. It may perhaps be argued whether HITT is a true auto-immune condition, since this requires the drug heparin to be present as a cofactor. However, there are several homologues to HITT, and there exist several in vivo heparin-like molecules. One homologue is the condition some workers call spontaneous HIT, and which we call HIT-like syndrome [65,66]. This is the condition where autoantibodies are formed against PF4 without a patient’s prior exposure to heparin. In such conditions, precipitation of the event is usually associated with recent surgery (especially knee surgery) or infection. It is thought that natural heparin-like molecules (including chondroitin sulfate and polyphosphates) can take the place of heparin, and lead to generation of autoantibodies against PF4/chondroitin sulfate or PF4/polyphosphate, or perhaps no additional polyanion for pathogenicity, leading to appearance of autoantibodies against PF4.

Another associated anti-PF4 related condition occurs as a rare adverse event following vaccination with adenovirus-based vaccines. This condition, called VITT, has been recently highlighted to occur following vaccination with some adenoviral-based COVID-19 vaccines, such as those manufactured by AstraZeneca (ChAdOx1-S/Vaxzevria) and Johnson & Johnson/Janssen (Ad26.COV2.S/Jcovden), which are otherwise aimed to reduce the incidence and morbidity of COVID-19 [65]. This condition may occur in approximately 1 in 50,000 doses of vaccine, and mimics HITT and HIT-like syndrome, since patients generate autoantibodies against PF4 that leads to platelet activation through Fc binding [63,64,65,66,67,68,69]. However, the epitopes on PF4 to which the VITT antibodies bind are different to those in HITT and HIT-like syndrome, and whether the antibodies bind directly to PF4, or if there is an associated PF4 binding factor, is unknown. However, if there is an associated PF4 binding factor in VITT, it is not heparin, and indeed, heparin can act to interfere with (even displace) the binding of the autoantibodies to PF4 [63,64,69]. It also remains to be determined whether this condition may be triggered by the adenoviral vector or by viral components encoded by the transfected genetic material, especially the SARS-CoV-2 spike protein which is prothrombotic by itself.

### 4.3. Acquired Immune Thrombotic Thrombocytopenia

Another autoimmune condition that causes thrombosis is acquired immune thrombotic thrombocytopenic purpura (iTTP) [70,71,72]. This is a rare and potentially lethal disease characterized by fragmentary hemolysis, moderate-to-severe thrombocytopenia, end-organ dysfunction, and severely reduced levels (<10%) of ADAMTS13 (A Disintegrin-like Metalloprotease domain with ThromboSpondin type 1 motifs). Survival in iTTP has improved significantly since introduction of plasma exchange as standard therapy combined with immune suppression to address the underlying pathophysiology. A host of challenges remain, however, including prompt recognition of disease, treatment of end-organ effects of disease, improving the early mortality rate, significantly reducing the relapse rate as well as addressing refractory disease. Identification of iTTP requires both recognition of very low levels of ADAMTS13, and also an immune basis for the deficiency [72,73,74]. Testing for ADAMTS13 antibodies can be undertaken by various means, although the majority of cases appear to be inhibitory antibodies, thus potentially detectable by Bethesda-like assays (similar to that discussed previously for acquired hemophilia A).

## 5. Autoantibodies Interfering with Hemostasis in COVID-19 and after Vaccination against COVID-19

COVID-19 represents a contemporary and paradigmatic example of the potential for generation of autoantibodies that may interfere with hemostasis, and which may lead to either bleeding or thrombosis, although the latter type predominates. We have already mentioned VITT, which arises following immunisation with adenovirus-based vaccines against COVID-19, as well as vaccine associated acquired hemophilia A. However, that represents only a segment of the auto-immune events potentially occurring as a result of COVID-19 or vaccination against COVID-19. For example, there have been multiple reports of association of COVID-19 with the presence of aPL, either solid phase and/or LA [75,76,77]. One difficulty here, however, is providing evidence of causal association vs. chance association. For example, in a recent review of the literature, we identified numerous reports of this association, but also many confounders [75,76]. The identification of APS requires both the presence of clinical evidence of thrombosis or pregnancy/fetal morbidity, as well as laboratory evidence of presence of aPL. For the former, thrombosis is already a feature of severe COVID-19 through multiple pro-thrombotic mechanisms [78,79,80,81,82], so this confounds the clinical evidence required to identify APS. For the latter, aPL are present in a proportion of asymptomatic patients (potentially up to 5% of the general population may be positive in one or more aPL tests). Thus, aPL may be present in COVID-19 patients, just as a chance event. Second, many patients with moderate to severe COVID-19 are given anticoagulant therapy to treat and/or prevent thrombosis [83,84,85,86]. Typically, this treatment encompasses heparin (for short term, hospital patients), or possibly one of direct oral anticoagulants (DOACs) (for longer term, post hospital discharge). In turn, heparin or DOAC presence may interfere with LA testing and yield false positive LA findings [61,87]. Third, for diagnosing APS, it is important to identify the persistence of these aPL, with this assessed using testing on a repeat sample some 12 weeks apart [57,58]. Most of the COVID-19 patients reported to have aPL in the literature were never repeatedly tested for confirmation, and thus do not strictly fit the criteria for definite APS [75,76]. In addition, at least one of these aPL testing events, potentially both, will be in patients anticoagulated for prevention or treatment of thrombosis. Again, all these anticoagulants can interfere with LA testing, and lead to false positive aPL determination. Additionally, relevant is that the prevalence of reported aPL/APS in COVID-19 varies widely in the literature, and up to 70% (for LA positivity) in some reports, although not necessarily associated with thrombosis [75,76,77,88]. Even if aPL are persistent (a requirement for identification of APS), these may not be associated with worse clinical outcomes, as recently reported in a prospective cohort of hospitalised SARS-CoV-2 (severe acute respiratory syndrome coronavirus 2) infected patients [89].

Perhaps unsurprisingly, COVID-19 has been associated with the majority of autoimmune conditions described in the previous sections of this review. In addition to potential APS, COVID-19 has been associated with HITT and HIT-like syndrome [64,90,91], as well as with acquired hemophilia A [92] and also acquired FV deficiency [93]. Again, the difficulty here is identifying if COVID-19 contributes to the reported events (i.e., “actor”), or whether COVID-19 is simply coincidental (i.e., “bystander”). Certainly, it is plausible that COVID-19 may contribute to these events, since SARS-CoV-2 (the virus responsible for COVID-19) causes major disruption to normal physiological processes, fostering a prothrombotic, hyper-inflammatory condition [94,95,96]. Indeed, many autoantibodies are reportedly associated with COVID-19 (Table 3), some of which could feasibly contribute to the prothrombotic milieu that is COVID-19. To some extent, what happens in COVID-19 may also apply to other infectious diseases. For example, Rivera-Correa and Rodriguez compared the development of autoantibodies in COVID-19 to that of malaria, and drew many parallels [97]. Similarly, it is likely that in COVID-19, as in other infectious diseases, molecular mimicry, a hyperactive immune system, and the virus itself represents the triad of plausible causal auto-immune events [91,98]. Other auto-immune conditions related to hemostasis and associated with COVID-19 include ITP [99,100,101] and TTP [102,103]. In terms of the latter, a large body of data indicates that COVID-19 is often associated with ADAMTS13 deficiency [104,105,106]; however, this may often be due to exhaustion of ADAMTS13 reserves due to the associated elevated release of VWF, rather than any immune basis or TTP. Thus, COVID-19 may also represent a form of secondary thrombotic microangiopathy [106], and mild loss of ADAMTS13 is more likely than is acquired TTP [104] in COVID-19.

Similarly to what occurs in COVID-19, vaccination against COVID-19 is also associated with a variety of autoimmune events, including those previously mentioned, as well as others, with some of these potentially associated with bleeding or thrombosis risk. For example, one recent case-based review identified 16 cases associated with inflammatory arthritis, SLE, vasculitis, Jo1 syndrome, Guillain-Barré syndrome, myocarditis [107]. Happaerts et al. described a case of acquired hemophilia A post vaccination, and reviewed 17 other cases reported in the literature [31]. Of interest, unlike VITT, which is associated with the use of adenovirus vaccines, most cases of acquired hemophilia A have been associated with mRNA-based vaccines (Pfizer [BNT162b2/Comirnaty] and Moderna [mRNA-1273/Spikevax]) [29]. Like COVID-19 above, vaccination against COVID-19 has also reportedly associated with ITP [108,109] and TTP [110].

In summary, both COVID-19 and vaccination against COVID-19 may trigger similar pathways to development or exacerbation of autoimmune diseases [111], and both may be associated with development of various autoimmune events [112,113] in addition to those we have described here.

## 6. Conclusions

The immune, complement and hemostasis pathways are intricately connected. Dysfunction in one of the pathways may affect one of the other pathways. In the current narrative review, we have focussed on the link between auto-immunity and hemostasis dysfunction. In turn, hemostasis dysfunction may manifest as either bleeding or thrombosis, depending on what type(s) of antibodies are generated. Antibodies may be generated against procoagulant clotting proteins, either leading to their clearance from circulation, or inhibiting their function, with development of bleeding diathesis, such as in acquired hemophilia A or AVWS. Alternatively, antibodies may be generated against components of the hemostasis pathways that act to moderate procoagulant activity. An example here is represented by antibodies against ADAMTS13, leading to clearance of this protein or reduction in activity, and resultant excess of VWF that promotes a thrombotic milieu. In the most severe cases, ADAMTS13 activity approaches 0%, and acquired TTP is evident. Another example is generation of antibodies against heparin in complex with PF4, leading to HIT, or analogous situations that do not require presence of heparin (i.e., HITT-like syndromes, VITT). In these cases, platelets can activate, leading to platelet aggregation, clearance of platelets and resultant thrombosis. Alternatively, other antibodies can be generated against various platelet glycoproteins, such as in ITP, and lead to platelet clearance, without activation, and this thus leads to thrombocytopenia and potential bleeding.

Thus, there is a complex interplay at work. For example, everyone is at risk of COVID-19, and most people have been vaccinated against COVID-19. This general population comprises both healthy and unwell individuals, or patients with cancer, or APS, or other pathology. Thus, a patient with cancer may get COVID-19, and this event may magnify their risk of thrombosis, or of acquiring an autoimmune condition. Here, there may be an unfortunate synergy, so that the final pathology may be more than the sum of its parts.

## Figures and Tables

**Table 1:** Some key procoagulant and anticoagulant components of hemostasis.

‘Procoagulant’	‘Anticoagulant’
Platelets	Protein C
Clotting factors (fibrinogen [I], II, V, VII, VIII, IX, X, XI)	Protein S Antithrombin
Factor XIII	Tissue-Factor Pathway Inhibitor
Thrombin (activated FII)	ADAMTS13
von Willebrand factor (VWF)	Endothelial cells
Factor V mutations (e.g., Leiden)	Heparin-like molecules
Phospholipids, CaCl_2_	
ADP, ATP	

**Table 2:** Summary of major autoimmune conditions associated with bleeding or thrombosis.

Conditions Associated with Bleeding	Conditions Associated with Thrombosis
Acquired hemophilia A (i.e., autoantibodies against FVIII)	Antiphospholipid (antibody) syndrome (APS)
Autoantibodies against other coagulation proteins	Heparin induced thrombotic thrombocytopenia (HITT)
Acquired von Willebrand syndrome (AVWS; only a proportion of AVWS are autoimmune related)	Autoimmune thrombotic thrombocytopenia (HIT-like)
Acquired autoimmune thrombocytopenia (ITP)	Vaccine induced (immune) thrombotic thrombocytopenia (VITT)
	Acquired autoimmune thrombotic thrombocytopenia (iTTP)

**Table 3:** Some autoantibodies associated with COVID-19, or due to vaccination against COVID-19.

Reported Autoantibodies	Association with Derangement of Hemostasis
Antiphospholipid antibodies (aPL)	Antiphospholipid (antibody) syndrome (APS) (thrombosis)
Autoantibodies against platelet factor 4 (PF4)	Heparin induced thrombotic thrombocytopenia (HITT) (thrombosis) Autoimmune thrombotic thrombocytopenia (HIT-like) (thrombosis) Vaccine induced (immune) thrombotic thrombocytopenia (VITT) (thrombosis)
Autoantibodies against ADAMTS13	Acquired autoimmune thrombotic thrombocytopenia (iTTP) (thrombosis)
Autoantibodies against factor VIII (FVIII)	Acquired hemophilia A (bleeding)
Autoantibodies against other factors	Various acquired factor deficiencies (generally bleeding)
Autoantibodies against platelets	Acquired autoimmune thrombocytopenia (iITP) (bleeding)

## Data Availability

Not applicable.
